# Design and Characterization of In-One Protease-Esterase PluriZyme

**DOI:** 10.3390/ijms232113337

**Published:** 2022-11-01

**Authors:** Laura Fernandez-Lopez, Sergi Roda, Jose L. Gonzalez-Alfonso, Francisco J. Plou, Víctor Guallar, Manuel Ferrer

**Affiliations:** 1 Department of Applied Biocatalysis, ICP, CSIC, 28049 Madrid, Spain; 2 Department of Life Sciences, Barcelona Supercomputing Center (BSC), 08034 Barcelona, Spain; 3 Institution for Research and Advanced Studies (ICREA), 08010 Barcelona, Spain

**Keywords:** esterase, *PluriZyme*, protease, protein engineering, computational chemistry, cascade reaction

## Abstract

Proteases are abundant in prokaryotic genomes (~10 per genome), but their recovery encounters expression problems, as only 1% can be produced at high levels; this value differs from that of similarly abundant esterases (1–15 per genome), 50% of which can be expressed at good levels. Here, we design a catalytically efficient artificial protease that can be easily produced. The *PluriZyme* EH_1AB1_ with two active sites supporting the esterase activity was employed. A Leu24Cys mutation in EH_1AB1_, remodelled one of the esterase sites into a proteolytic one through the incorporation of a catalytic dyad (Cys24 and His214). The resulting artificial enzyme, EH_1AB1C_, efficiently hydrolysed (azo)casein at pH 6.5–8.0 and 60–70 °C. The presence of both esterase and protease activities in the same scaffold allowed the one-pot cascade synthesis (55.0 ± 0.6% conversion, 24 h) of L-histidine methyl ester from the dipeptide L-carnosine in the presence of methanol. This study demonstrates that active sites supporting proteolytic activity can be artificially introduced into an esterase scaffold to design easy-to-produce in-one protease-esterase *PluriZymes* for cascade reactions, namely, the synthesis of amino acid esters from dipeptides. It is also possible to design artificial proteases with good production yields, in contrast to natural proteases that are difficult to express.

## 1. Introduction

One-pot cascade reactions are chemical processes highly appealing to the industrial sector, as they allow the synthesis of complex products, starting from relatively simple reaction conditions [1,2,3,4]. Nevertheless, the implementation of these reactions is a demanding task, and usually, it requires the engineering of each catalyst from each internal chemical reaction. Moreover, catalysts must be specific for their reactant, avoiding unpleasant side products as much as possible. For that reason, enzymes are excellent candidates to set up multistep tandem reactions, since they work under milder conditions than inorganic catalysts and are also regio- and stereo-selective/specific [2,3,4]. Additionally, recent advances in the rational design and directed evolution of enzymes have introduced a significant success rate in improving diverse enzymatic properties, allowing them to compete with conventional catalysts [2,3,4,5]. Remarkably, current developments include designing enzymes with new-to-nature catalytic activities, such as carbene transfer in an engineered cytochrome P450 enzyme [6], expanding the variety of the cascade reactions we can tackle. Another interesting example of the usage of enzymes for cascade reactions is the one developed by Merck & Co., to synthesize islatravir (a potential drug for HIV treatment) from relatively simple building blocks [7].

Performing the cascade reaction within a single enzyme introduces complexities. For this reason, the study and design of biocatalysts performing different chemical reactions in either the same protein scaffold [8,9,10,11,12,13] or by linking multiple domains [14,15,16,17] have become a hot topic in protein engineering through a number of strategies [18,19]. One of these strategies is to benefit from the capabilities of protein engineering supported by the computational resources for the design of artificial enzymes with a superior or novel performance, compared to natural enzymes. Beyond the engineering of enzymes by these techniques, the possibility of incorporating active centers into protein scaffolds opens the opportunity to design artificial biocatalysts. A number of computational methods have been successfully applied to introduce biological active sites into protein scaffolds. They include, the Rosetta-like methods and the Protein Energy Landscape Exploration (PELE) software, through which biological sites supporting ester-hydrolysis have been incorporated in different protein scaffolds [8,9,19]. However, PELE is the only described method to generate several artificial biological actives sites in the same enzyme scaffold, which open a range of possibilities in cascade reactions, as demonstrated using different enzyme scaffolds into which two same or different biological activities were introduced [8,20]. These two-active sites enzymes, named *PluriZymes*, could introduce an ideal scenario for one-pot cascade reactions, by reducing the costs of producing two enzymes separately. In some cases, their use may also allow increased yields by facilitating the transfer of reaction intermediates between active sites within the same protein, compared to the transfer between sites of different proteins [20]; however, this will depend on the architecture and positioning of these sites.

One example is our recent work building *PluriZymes* in two different enzyme families. First, we developed an esterase, EH_1AB1_, where a second catalytic triad (Ser-His-Asp/Glu) was added, creating an enzyme with artificial and native active sites supporting ester hydrolysis (*T*_opt_ of 8–45 °C) [8]. Importantly, the native active site could be transformed into a metal-complex chemocatalytic site by adding a suicide inhibitor, allowing the oxidation and Friedel–Crafts alkylation reactions. Thus, one-pot cascade reactions could be constructed from this biocatalyst [8]. Second, we took an ω-transaminase and added an artificial site supporting ester hydrolysis through the introduction of a catalytic triad (Ser-His-Asp/Glu). A polypeptide having two biotic sites catalysing different types of chemical reactions was thus designed, which could transform oxo-esters into amino acids in a one-pot reaction [20].

In the present study, we aimed at a different approach, pushing the limits of a hydrolase site by adding extra biochemistry through the addition of a cysteine-histidine catalytic dyad. Using our *PluriZyme* EH_1AB1_, we introduced the protease activity by designing a single mutant, Leu24Cys, which was capable of recycling a histidine residue, His214, from an already existing catalytic triad. A priori, computational analyses indicate that the mutation should not disrupt the esterase activity of the recycled esterase site. Therefore, the newly designed *PluriZyme*, herein referred to as EH_1AB1C_, included three potential sites. The first supports ester hydrolysis through a native catalytic triad (Ser161, Asp256 and His286) and an oxyanion hole (Gly88, Gly89 and Gly90), with Ser161 being the nucleophile. The second, also supporting ester hydrolysis, would employ an artificial catalytic triad (Ser211, Asp25 and His214) with Ser211 as the nucleophile and an oxyanion hole (Gly207, Tyr208 and Phe209). The third would support the protease activity through a catalytic dyad (Cys24 and His214).

Adding a site supporting proteolytic activity was targeted, as proteases are pivotal enzymes for the hydrolysis of peptide bonds in materials where proteins are abundant components and are also widely used in organic synthesis [21]. This is why they constitute 60–65% of the global industrial market, growing at an annual growth rate of 5.6% [22]. Through evolution, proteases have adapted to the wide range of conditions found in complex organisms (variations in pH, reductive environment, etc.) and use different catalytic mechanisms for substrate hydrolysis [23]; their mechanism of action classifies them as either serine, cysteine or threonine proteases (amino-terminal nucleophile hydrolases) or aspartic, metallo and glutamic proteases (with glutamic proteases being the only subtype not found in mammals thus far) [24]. Proteases specifically cleave protein substrates either from the N or C termini (aminopeptidases and carboxypeptidases, respectively) and/or in the middle of the molecule (endopeptidases). Proteases can be easily screened by functional screens or in silico predictions in microorganisms or microbial communities by applying genomic and metagenomic approaches [22]. Of course, it should be stressed that the novelty itself does not guarantee a better enzymatic performance and better opportunities for commercialization, whose analysis requires laborious wet lab work. This is not a trivial exercise, given that not all genes in a genome or a metagenome can be successfully cloned and expressed. This is especially important in the cases of proteases that suffer major problems of expression, compared to other types of enzymes that are as easy to be screened as proteases but better to be produced at high levels [22]. Proteases are used in a broad range of applications, including biorefineries targeting a broad range of biomasses [22]. However, the known and new proteases positively impact additional processes, such as cascade reactions where proteases have a pivotal role [25]. Here, we targeted a cascade reaction involving peptide bond cleavage and ester bond formation and demonstrated that the newly designed EH_1AB1C_ *PluriZyme* was capable of converting the dipeptide L-carnosine (β-alanine-L-histidine) into L-histidine methyl ester, an intermediate for the design of Schiff base ligands [26].

## 2. Results

### 2.1. Molecular Simulations

**Figure 1:**
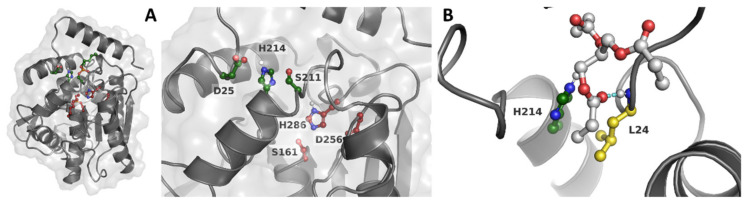
(**A**) Slice of the 3D structure of EH_1AB1_ representing the main and artificial active sites. The C atoms are stained in maroon and dark green in the main active site and artificial active site, respectively. (**B**) Representative binding pose of glyceryl tripropionate (with C atoms stained in gray) where we can see the position of Leu24. Leu24 (in yellow) forms a hydrogen bond with the carbonyl O atom in the ester bond by the NH group in the backbone (indicated by a cyan dashed line) and is close to the His214 residue.

This work is based on the design of *PluriZymes* (latin root pluri: multiplicity), an enzyme design in which a single polypeptide harbours two different active centers, one native and one artificial [8,20]. The idea is based on locating a native enzyme, through the Protein Energy Landscape Exploration (PELE) software, existing binding pockets where a target substrate could be accommodated and turning them into catalytic active sites by introducing all the residues needed for the catalysis. Following from this, we have recently successfully found and designed, by introducing a few mutations, a second artificial active site (Ser-His-Asp) in an esterase containing a native site to generate a *PluriZyme*, EH_1AB1_, with two efficient biological active sites for the ester hydrolysis that coexists in a close region. Compared to the natural triad (referred to as EH_1A1_), the newly introduced artificial site (referred to as EH_1B1_) is slightly more solvent exposed and located at an ~10 A distance (Figure 1A). We have considered increasing the number of chemical reactions within this enzymatic design. The computational protocol employed herein is similar to the one used in our *PluriZyme* designs, largely described in [8,9,19,27], but we have focused more on a local region exploration.

We first attempted to perform an additional global surface exploration for locating potential sites supporting the ester hydrolysis using glyceryl tripropionate as a probe. As shown in Figures S1 and S2, we did not obtain additional alternatives. We then considered the incorporation of a new active center to support another reaction, namely, proteolysis, to explore the possibility of designing an in-one protease-esterase *PluriZyme*. For this, we focused on the local analysis of the catalytic triad regions (Figure 1A), where we clearly observed how the more exposed site, EH_1B1_, could accommodate bulkier substrates (as in the case of peptides). Following the inspection of the best enzyme-substrate poses (Figure 1B), we decided to introduce the Leu24Cys variant, named EH_1AB1C_, which, together with the catalytic histidine of EH_1B1_, His214, could introduce a catalytic dyad similar to those seen in proteases.

**Figure 2:**
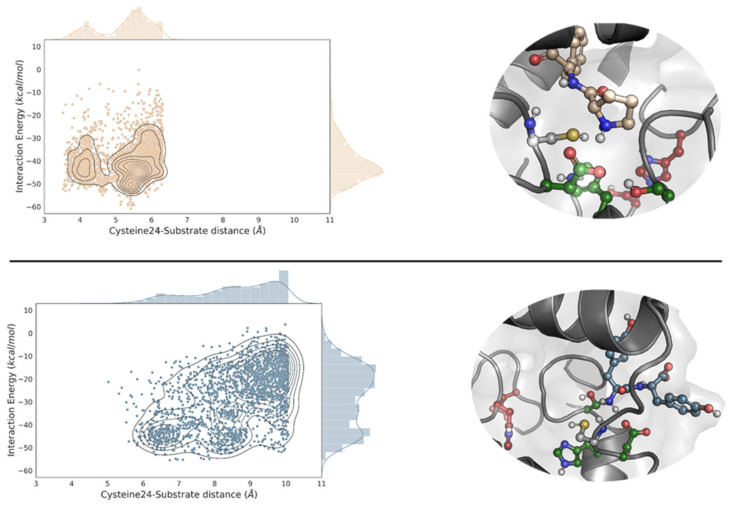
PELE’s energy analysis and representative geometries. EH_1AB1C_ density plots of the distribution of the catalytic cysteine-substrate distances against the interaction energy for the best-bound dipeptide substrate (PF, top) and the worst one (YY, bottom). Only the 10% lowest percentile, regarding the distance is shown. In the right panel, we display representative catalytic poses of the dipeptide substrate in the putative protease site. The C atoms are stained in maroon, dark green and yellow in the EH_1A1_ active site residues, EH_1B1_ and Cys24, respectively, and each substrate is shown using the density plot colour (in wheat for PF and blue for YY). The energy profiles were created with the Matplotlib library [28].

We proceeded by preparing multiple dipeptides, namely, AH, AQ, DI, EA, FF, KA, LA, LL, NV, PF, QQ, RG, SW, TM, YN and YY, and performed a local PELE exploration for each dipeptide. By doing so, we can model the propensity to form catalytically active positions between each peptide bond and the newly designed catalytic dyad: Cys24 and His214. Here, we were not searching for the best peptide but to see if a diverse set of them could reach catalytically active conformations. Interestingly, as shown in Figure 2, Table S1, and Figures S3–S5, all dipeptides, except for YY were able to find the EH_1B1_ site and reach catalytic poses around the engineered cysteine-histidine dyad. Likewise, it can be seen that the catalytic hydrogen bond distances (Cys to His) were quite good during the simulation (Table S2, Figures S6 and S7).

### 2.2. Experimental Validation: EH_1AB1C_ Is an Efficient Protease

**Figure 3:**
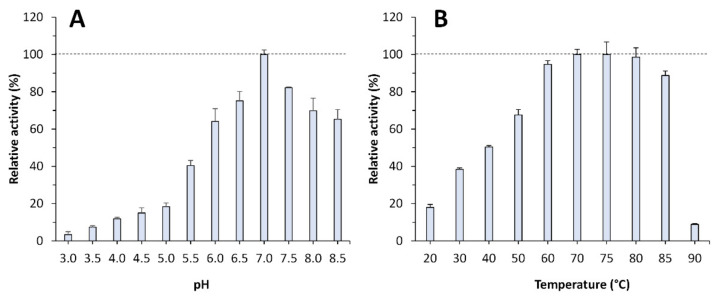
pH and temperature profiles of the purified EH_1AB1C_. (**A**) pH profile at 30 °C. (**B**) Temperature profile at pH 7.0. The maximal activity was defined as 100%, and the relative activity is shown as the percentage of the maximal activity (mean ± SD of triplicates), determined under standard reaction conditions with BODIPY-FL-casein (in panel (**A**)) and azocasein (in panel (**B**)) as the substrates. The graphics were created with Excel version 14.0.

The recombinant mutant, hereafter referred to as EH_1AB1C_, was successfully expressed in soluble form in *Escherichia coli* and purified by nickel affinity chromatography. The purified protein (approx. 10 mg per litre of culture) was desalted by ultrafiltration, and its proteolytic activity was tested through a general fluorescence assay, namely, the BODIPY-FL-casein assay using the EnzChek^®^ Protease Assay Kit, which is insensitive to pH changes. First, the pH profile of the enzyme was obtained (Figure 3A). Its optimal pH for activity was 7.0, retaining more than 70% of the maximal activity at pH values from 6.5 to 8.0. We then analysed its temperature profile using the chromogenic substrate azocasein. At pH 7.0, EH_1AB1C_ showed maximal activity at 70–75 °C, retaining more than 70% of the maximum activity at 50–85 °C (Figure 3B). The specific activity of EH_1AB1C_ was compared to that of the commercial protease Neutrase 0.8 L (Novozymes A/S, Bagsvaerd, Denmark). At pH 7.0 and 30 °C, the specific activity of EH_1AB1C_ was 2.63 ± 0.06 U/mg protein, while that of the commercial Neutrase 0.8 L was 1.86 ± 0.11 U/mg protein. Note that the original design, EH_1AB1_, did not show any proteolytic activity with BODIPY-FL-casein or azocasein, demonstrating that the incorporation of the dyad Cys24-His214 introduced proteolytic activity.

The esterase activity of the EH_1AB1C_ mutant was quantified at 30 °C and pH 8.0 with the model ester glyceryl tripropionate and compared to that of the initial construct EH_1AB1_. This substrate can be converted by both esterase sites (the native and the artificial sites). We found that the EH_1AB1C_ mutant is an efficient esterase capable of hydrolysing this ester at 3160 ± 76 U/g. However, we observed that this value was only 14.5% of that of EH_1AB1_. To evaluate whether this reduction could be because the artificial esterase center (B site), during its remodelling to add a protease center, has been altered and with it, its activity, would require evaluating the activity with B-site specific esters. However, all tested esters hydrolysed by this site are also hydrolysed by the native esterase center (A-site), due to the broad substrate specificity of the latter [8]. Therefore, we cannot be sure whether the observed reduction in activity is due to a possible effect of the mutation introduced (Leu24Cys) on the architecture of the B-center, to a local effect on the structure, or to the possibility of partial autolysis (self-digestion) by the addition of the protease site.

### 2.3. Application of EH_1AB1C_ in a One-Pot Cascade Reaction

**Figure 4:**
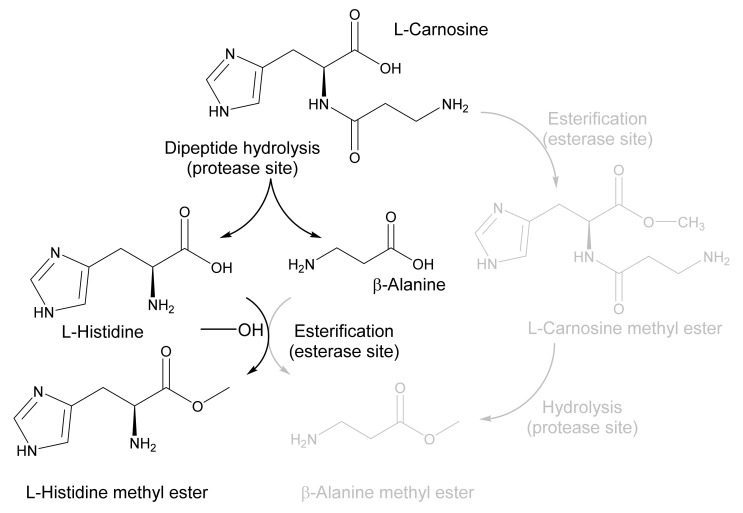
Schematic representation of the main product obtained in a one-pot reaction with the dipeptide L-carnosine (β-alanine-L-histidine) and methanol. A schematic representation of the two reaction intermediates and the two possible products (β-alanine and L-histidine methyl esters) is shown. As shown in the figure, the preferential route found is the production of L-histidine methyl ester via the hydrolysis of L-carnosine at the protease site and the selective esterification with methanol of L-histidine (but not β-alanine) at the esterase site. No appreciable formation of L-carnosine methyl ester and β-alanine methyl ester (gray colour) was detected. The figure was created using ChemDraw 18.2.

Following the computational design of a protease site in EH_1AB1_ and the characterization of the successful variant EH_1AB1C_, we wanted to test the ability of this validated *PluriZyme* to catalyse a cascade reaction of interest. As a model reaction, we chose to synthesize L-histidine methyl ester, an intermediate for the design of Schiff base ligands [26], from the dipeptide L-carnosine (β-alanine-L-histidine) (Figure 4).

**Figure 5:**
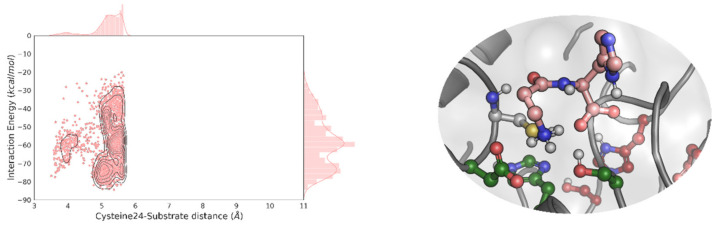
EH_1AB1C_ density plot of the distribution of the catalytic cysteine-substrate distance against the interaction energy for L-carnosine. Only the 10% lowest percentile, regarding the distance is shown. In the right panel, we display the representative catalytic poses of L-carnosine in the putative protease site. The C atoms are stained in maroon, dark green, and yellow in the EH_1A1_ active site residues, EH_1B1_, and Cys24, respectively, and L-carnosine is shown using the pink colour used in the density plot. The energy profiles were created with the Matplotlib library [28].

**Figure 6:**
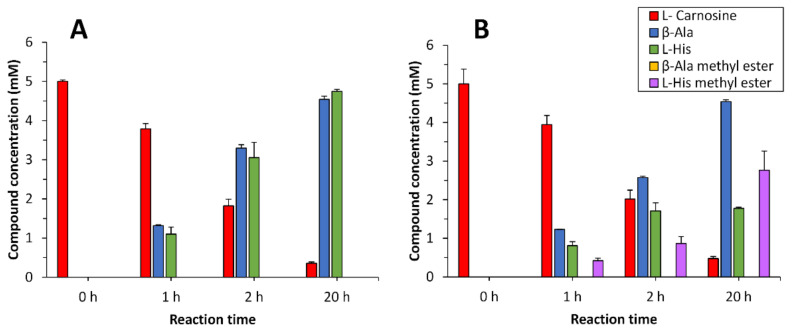
Concentrations of the substrates, intermediates and final products obtained for the conversion of L-carnosine by the EH_1AB1C_ *PluriZyme* in the absence (**A**) or presence (**B**) of methanol. Reaction conditions in A: [cells expressing EH_1AB1C_]: 5 μL of resuspended cells at OD_600 nm_ of 15.0; [L-carnosine]: 5 mM; buffer: 92.5 µL 40 mM HEPES, pH 7.0; reaction final volume: 100 μL. Reaction conditions in A: [cells expressing EH_1AB1C_]: 5 μL of resuspended cells at OD_600 nm_ of 15.0; [L-carnosine]: 5 mM; solvent: 92.5 µL methanol; reaction final volume: 100 μL (92.5% methanol). Note, that the amount of the β-Ala methyl ester was below the detection limit under our experimental conditions, and this is why yellow bars are not visible in the figure. The figure was created using SigmaPlot 14.0 software.

The simulation of the reaction by a local PELE exploration showed efficient L-alanine-L-histidine (AH) catalytic (hydrolytic) binding poses at the proteolytic site (Table S1). We also obtained good catalytic poses for L-carnosine (β-alanine-L-histidine) (Figure 5; Table S1). Thus, we expected that L-carnosine would be hydrolysed at the protease site, due to its similarity with the L-alanine-L-histidine. We set up two reactions at 40 °C and pH 7.0. The first one contained L-carnosine (5 mM) in the buffer, 40 mM HEPES, at pH 7.0. The second one represented a one-pot cascade reaction with all the reagents necessary for the hydrolysis of L-carnosine and the esterification of the corresponding reaction products with methanol, i.e., L-carnosine and methanol. Following the addition of the *E. coli* cells expressing the EH_1AB1C_ *PluriZyme*, the levels of the substrate L-carnosine, the intermediates β-alanine and L-histidine, and the possible products β-alanine methyl ester and L-histidine methyl ester, were quantified using high-performance liquid chromatography (HPLC) over 20 h (Figure 6 and Figure S8). Note that for these tests, cells expressing EH_1AB1C_ were used instead of purified protein to increase the stability of the biocatalysts, which in soluble form may be inactivated.

We found that the L-carnosine dipeptide (concentration of 5 mM) was fully converted (>93.0 ± 0.1%) after 20 h in the buffer, obtaining β-alanine (4.5 ± 0.1 mM) and L-histidine (4.75 ± 0.05 mM) (Figure 6). When the reaction was performed in methanol, L-carnosine was converted after 20 h (90.5 ± 12.3%), with the main products being β-alanine (4.5 ± 0.1 mM), L-histidine (1.8 ± 0.1 mM) and L-histidine methyl ester (2.8 ± 0.5 mM). β-Alanine methyl ester and L-carnosine methyl ester were not found as products. This result demonstrates that L-carnosine is hydrolysed at the artificial proteolytic site; furthermore, the esterase site only recognized L-histidine but not β-alanine as a substrate, which is esterified to yield L-histidine methyl ester as the only amino acid ester.

To highlight, we found that when using as biocatalyst cells expressing the original EH_1AB1_ *PluriZyme*, L-carnosine was not converted to any of the intermediates or final products (Figure S9A); this indicates that the proteases in *E. coli* do not affect the results and that in the absence of the artificial proteolytic center, the cascade reaction is not feasible. Further, both the original EH_1AB1_ *PluriZyme* and the mutant EH_1AB1C_ *PluriZyme* biocatalysts were shown to convert L-histidine to L-histidine methyl ester in the presence of methanol, but not β-alanine to the corresponding methyl ester (Figure S9B,C). These control tests, together with the results presented in Figure 6, demonstrated that the incorporation of the dyad Cys24-His214 in the EH_1AB1C_ *PluriZyme* was responsible for the proteolysis of L-carnosine, and that the esterase(s) active site(s) originally present in EH_1AB1C_ supported the acylation of L-histidine and the production of the amino acid ester (L-histidine methyl ester).

## 3. Discussion

Proteases that can break ester bonds and esterases and lipases that can break amide bonds exist in nature; they are an example of catalytically promiscuous enzymes [29,30,31,32,33,34]. The presence of a Ser or a Cys catalytic residue, along with differences in the substrate binding site, seems to be the main reason for substrate discrimination and the promiscuous behavior. When attempting to engineer such activity from scratch (ab initio) or when introducing enough mutations to accommodate a dyad/triad, we typically find low activity profiles [35,36,37]. Nevertheless, these results have paved the way for more recent implementations, such as our recent *PluriZymes*, where the extensive in silico optimization (catalytic distances) has provided wide substrate promiscuity, high substrate conversion rates, and even the development of cascade reactions [8,9]. In this study, we introduced the protease activity by reusing a (previously engineered) esterase site. We recycled the catalytic histidine and inserted only a cysteine residue, achieving high catalytic rates in the standard peptide assays. As in our previous designs, the potential of running accurate enzyme-substrate induced fit simulations provided enough insights. Thus, these results again illustrate how simulation techniques using molecular modelling are mature enough and capable of providing a realistic description of localized changes. Such modelling potential, along with the propensity of catalytic dyads/triads to form (stabilizing) hydrogen bonds, makes the design of hydrolase active sites an affordable task; we have succeeded in all recent attempts [8,9,20].

The main reason behind our efforts derives from our goals of developing single scaffold enzymes with multiple biochemical activities that are capable of performing one-pot cascade reactions. Previous attempts in the native esterase EH_1_ focused on introducing a second artificial esterase active site and the incorporation of an irreversible-linked inhibitor containing a meta-chelating moiety to one of the sites, allowing the addition of artificial abiotic oxidative chemistry as a complement to the original biotic esterase activity [8]. More recently, we were able to add artificial biotic esterase activity into an ω-transaminase [20]. With our new catalytically efficient design, EH_1AB1C_, we are now capable of breaking amide bonds and forming ester bonds to yield natural and nonnatural amino acid esters, herein exemplified by L-histidine methyl ester, which are important intermediates in organic synthesis [38]. Amino acid methyl esters, such as L-histidine methyl ester, can be chemically synthesized at room temperature through the esterification of L-histidine with trimethylchlorosilane (TMSCl) and methanol at high yields (88–96%, using 10 mM L-histidine) [38]; the biocatalysts reported herein offer milder and environmentally friendly conditions and good yields for the synthesis of amino acid esters useful in the pharmaceutical industry. In addition, both protease and esterase sites in EH_1AB1C_ may offer specificity, as shown here, for example, by the capacity of the latter to esterify L-histidine but not β-alanine. The capacity of some proteases to catalyse ester hydrolysis has been used to produce amino acid esters with a high optical purity through the selective hydrolysis of D,L-amino acid esters (e.g., methyl and benzyl esters) to provide, for example, L-amino acids and optically pure D-amino acid esters [39,40]. However, in this case, an amino acid ester is needed as the initial substrate, in contrast to our *PluriZyme*, which can directly use dipeptides and possibly longer oligopeptides.

We would like to highlight that the reaction conditions for the substrate reported in this work and others yet to be tested have not been optimized. Thus, this design offers interesting properties to be utilized in industrial settings, such as an integrated biorefinery for biomass recovery based on proteases for the production of protein hydrolysates, bioactive peptides, and amino acids applicable to a wider range of applications. It also offers a platform to synthesize a wide range of biobased products through in-one cascade reactions involving ester and amine bond hydrolysis and formation.

## 4. Materials and Methods

### 4.1. Materials

Azocasein (ref. A2765-1G), β-alanine (ref. 146064-25G), L-histidine (ref. H7750-25G), L-histidine methyl ester (ref. H15403-25G), β-alanine methyl ester (ref. 05210-10G), L-carnosine (ref. C9695-10MG), glyceryl tripropionate (ref. W328618-1KG-K), and methanol (ref. 34966-1L) were ordered from Merck Life Science S.L.U. (Madrid, Spain). The EnzChek^®^ Protease Assay Kit (ref. E6638) was provided by Invitrogen, Thermo Fisher Scientific Inc., Waltham, MA, USA. FMOC chloride (ref. GE3236-1G) was purchased from Glentham Life Sciences, Corsham, UK.

### 4.2. Protein and Chemical Preparation for the In Silico Analysis

The apo EH_1A_ crystal structure (5JD4) and the holo EH_1AB1_ crystal structure (6RB0) were prepared and protonated at pH 8.0, the pH at which the experimental assays were performed, using Protein Preparation Wizard [41]. This includes fixing side chains and loops missing in the crystal structure using Prime software [42]. The ester compound used as a probe to find noncatalytic hydrolase sites was glyceryl tripropionate; the peptide binding assays of the generated variant used 16 dipeptides (AH, AQ, DI, EA, FF, KA, LA, LL, NV, PF, QQ, RG, SW, TM, YN, and YY) as substrates. All substrates were modelled using the OPLS2005 force field [43]. The atomic charges of glyceryl tripropionate and the catalytic serine residues bound with the methyl hydrogen (*R*)-hexylphosphonate inhibitor [8,20] were calculated with Jaguar [44] using density functional theory with a B3LYP-D3 exchange-correlation functional and the polarized triple-zeta (pVTZ) basis set.

### 4.3. Protein Energy Landscape Exploration (PELE) Simulations

PELE was used to find the noncatalytic peptide binding sites in EH_1A_/EH_1AB1_ and check if the catalytic poses can be reached in the functionalized variant [8,9,19,27]. PELE is a Monte Carlo (MC)-based algorithm coupled with protein structure prediction methods [45]. The heuristic MC approach begins with the sampling of different microstates by initially applying small perturbations (translations and rotations) on the ligand. Then, the flexibility of the protein is taken into account by applying normal modes through the anisotropic network model (ANM) approach. Once the system has been perturbed, side chains of the residues near the ligand are sampled with a library of rotamers to avoid steric clashes. Finally, a truncated Newton minimization with the OPLS2005 force field [43] is performed, and the new microstate is accepted or rejected, according to the Metropolis criterion. The variable dielectric generalized Born non-polar (VDGBNP) implicit solvent [46] was applied to mimic the influence of water around the protein.

### 4.4. Prediction of ΔΔG in the EH_1AB1_ Variant

The ΔΔG(mut-WT) of stability in the experimentally tested variants was calculated using the module of thermodynamic stability from HotSpot Wizard, which uses FoldX to repair possible problems in the protein structure and Rosetta to perform the energy minimization and ΔΔG calculation (according to protocol 3 from Rosetta) [47].

### 4.5. Source and Production of EH_1AB1C_

The sequence of EH_1AB1C_ was synthesized by GenScript Biotech (GenScript Biotech, EG Rijswijk, Netherlands) and was codon-optimized to maximize the expression in *E. coli*. The gene was flanked by BamHI and HindIII (stop codon) restriction sites and inserted into a pET-45b(+) expression vector with an ampicillin selection marker (GenScript Biotech, EG Rijswijk, Netherlands), which was further introduced into *E. coli* BL21(DE3). The soluble N-terminal histidine (His)-tagged protein was produced and purified (98% purity, as determined by SDS–PAGE analysis using a Mini PROTEAN electrophoresis system, Bio-Rad, Madrid, Spain; Figure S10) at 4 °C after binding to a Ni-NTA His-Bind resin (Merck Life Science S.L.U., Madrid, Spain), as previously described [8,20], and stored at −20 °C until use at a concentration of 1.5 mg/mL in 40 mM 4-(2-hydroxyethyl)-1-piperazineethanesulfonic acid (HEPES) buffer (pH 7.0). Approximately 10 mg of purified EH_1AB1C_ was obtained on average from a 1-L culture.

### 4.6. Activity Tests

The proteolytic activity was tested with the EnzChek^®^ Protease Assay Kit containing casein labelled with BODIPY^®^ FL (E6638) dye (green fluorescence), which is insensitive to pH. The assay was prepared, according to the protocol provided by the manufacturer (Thermo Fisher, Madrid, Spain). If otherwise not indicated, the reactions were carried out in 96-well plates by adding 1 µL of BODIBY FL casein (1 mg/mL) to 99 µL of 40 mM HEPES buffer at pH 7.0 with 1.0–5.0 µg of protein and incubated for 1 h, protected from light at 30 °C. The fluorescence (λ_exc_: 485 ± 20 nm; λ_emm_: 528 ± 20 nm) was then measured in a Synergy HT multi-mode microplate reader (Biotek Instruments, Winooski, VT, USA). All values were determined in duplicate by determining the fluorescence at 60 min and were corrected for nonenzymatic transformation. For the pH determination (in triplicate), the previous conditions were used but with 50 mM Britton and Robinson buffer at pH 3.0–8.5. To determine the thermal profile (in triplicate), the substrate used was chromogenic azocasein. Briefly, to 25 µL of 2.5% *w/v* azocasein (prepared in water), 15 µL of 40 mM HEPES at pH 7.0 and 10 µL of EH_1AB1C_ (from a 1.4 mg/mL stock) were added. This reaction was incubated for 40 min with shaking at 700 rpm at different temperatures (20–90 °C). Then, 200 µL of trichloroacetic acid was added to stop the reaction, which was then centrifuged for 5 min at 13,000 rpm. Subsequently, 50 µL of this supernatant was added to 150 µL of 0.5 M NaOH in a 96-well plate, and its absorbance at 440 nm was measured in a Synergy HT Multi-Mode Microplate Reader. In all cases, the control reactions with EH_AB1_ under the same experimental conditions were performed. In all cases, the values were corrected for nonenzymatic transformation.

The hydrolysis of the model ester glyceryl tripropionate by EH_1AB1_ and EH_1AB1C_ was assayed using a pH indicator assay in 384-well plates (ref. 781162, Greiner Bio-One GmbH, Kremsmünster, Austria) at 30 °C and pH 8.0 (5 mM 4-(2-hydroxyethyl)-1-piperazinepropanesulfonic acid (EPPS) buffer containing 0.45 mM phenol red) in a Synergy HT multi-mode microplate reader in continuous mode at 550 nm over 30 min (extinction coefficient (ε) of phenol red: 8450 M^−1^ cm^−1^), as reported in [8,20]. The conditions for determining the specific activity (U/mg) were as follows: [protein]: 5 μg/mL; [ester]: 10 mg/mL; reaction volume: 44 μL; T: 30 °C; and pH: 8.0. The activity was calculated by determining the absorbance per minute from the slopes generated [8,20]. In all cases, all values in triplicate were corrected for nonenzymatic transformation, with the absence of activity defined as having at least a twofold background signal.

### 4.7. Cascade Reaction and the HPLC Analysis

L-Carnosine, used as the substrate, and β-alanine, L-histidine, β-alanine methyl ester and L-histidine methyl ester, used as the standards, were obtained from Merck Life Science S.L.U., Madrid, Spain. The reactions were performed using resting cell assays. Briefly, *E. coli* BL21(DE3) expressing EH_1AB1C_, His-tagged at the N-terminus, was grown at 37 °C on solid LB agar medium supplemented with 100 μg/mL ampicillin, and one colony was picked and used to inoculate 50 mL of Luria Bertani broth plus antibiotic in a 0.25-L flask. The culture was then incubated at 37 °C and 200 rpm overnight. Then, 50 mL of this culture was used to inoculate 1-l of LB medium plus antibiotic in a 2.5-L flask, which was then incubated to an OD_600 nm_ of approximately 0.7–1.0 at 37 °C. The protein expression was induced by adding isopropyl β-D-1-thiogalactopyranoside to a final concentration of approximately 1 mM, followed by incubation for 16 h at 16 °C at 220 rpm. The cells were harvested by centrifugation at 5000× *g* for 15 min to yield a pellet of 2–3 g/L (wet weight). The wet cell pellet was resuspended in 60 mL of 40 mM HEPES at pH 7.0 and centrifuged as before, and the cells were retained. Finally, the cells were resuspended in 60 mL of 40 mM HEPES at pH 7.0 until an OD_600 nm_ of 15.0 and used directly for the activity tests.

To evaluate the proteolytic capacity of EH_1AB1C_ against L-carnosine, the following reaction conditions were used in a 1.5 mL Eppendorf tube: [cells expressing EH_1AB1C_]: 5 μL of resuspended cells at OD_600 nm_ of 15.0; [L-carnosine]: 2.5 μL of a 200 mM stock solution in 40 mM HEPES at pH 7.0; T: 30 °C; buffer/solvent: 92.5 µL of 40 mM HEPES buffer at pH 7.0; and reaction final volume: 100 μL (100% buffer). To evaluate the capacity of EH_1AB1C_ to produce amino esters in a cascade reaction, the following conditions were used in a 1.5 mL Eppendorf tube: [cells expressing EH_1AB1C_]: 5 μL of resuspended cells at OD_600 nm_ of 15.0; [L-carnosine]: 2.5 μL of a 200 mM stock solution in 40 mM HEPES at pH 7.0; T: 30 °C; buffer/solvent: 92.5 µL of methanol; and reaction final volume: 100 μL (7.5% buffer and 92.5% methanol). Control reactions with L-histidine and β-alanine (using stock solutions at the same concentrations as L-carnosine) instead of L-carnosine were set-up. The same reaction tests were performed with cells expressing EH_AB1_ under the same experimental conditions.

Aliquots of 100 µL of the reaction mixture were taken at different times. The aliquots were filtered using 0.45 µm Durapore filter unit inserts (Millipore Corporation, Billerica, MA, USA) to remove the cells and stop the reaction. The solvent was evaporated using a SpeedVac Concentrator (Model 5301, Eppendorf, Hamburg, Germany) at 30 °C. The remaining L-carnosine and the formed reaction products were derivatized by adding 500 µL of B_4_Na_2_O_7_ buffer at pH 9.0 (100 mM) followed by the addition of 500 µL FMOC chloride (4 mM) in acetonitrile. The samples were shaken in a vortexer for 1 h. Then, the samples were analysed by HPLC using a quaternary pump (Model 600, Waters, Milford, MA, USA) coupled to an autosampler (Model 420, Varian ProStar, Wainut Creek, CA, USA). The injection volume was 10 µL. The column was a Zorbax Eclipse Plus C-18 (4.6 × 100 mM, 3.5 μm, Agilent Technologies, Santa Clara, CA, USA) at 40 °C, and the mobile phase was an acetonitrile/H_2_O gradient with both solvents acidified with 0.1% (*v*/*v*) formic acid and degassed with helium. The gradient (20 min in total) is specified as follows: 0–12 min, 20→90% acetonitrile; 12–15 min, 90% acetonitrile; 15–15.5 min, 90→20% acetonitrile; 15.5–20 min, 20% (initial conditions). The flow rate was 1.0 mL/min. The detection of peaks was carried out using a photodiode array detector (Model 335, Varian ProStar), and integration was carried out using Varian Star LC Workstation 6.41 at a wavelength of 264 nm. The quantification of the reaction products was performed with the calibration curves of the standards of L-carnosine and the corresponding amino acids and esters at concentrations between 0 and 500 µM.

## 5. Conclusions

To the best of our knowledge, we have designed the first example of an in-one protease-esterase *PluriZyme*. This construct was built by engineering an artificial active site supporting the protease activity into an esterase by remodelling one of the two esterase active sites of a recent *PluriZyme* design through a Let24Cys mutation. The resulting mutant does contain a native Ser-His-Asp catalytic triad supporting esterase activity and an artificial Cys-His dyad supporting proteolysis. We will continue to explore the biocatalytic potential of this new type of biocatalyst, as suggested by the data presented here, particularly for the synthesis of amino acid esters, which are versatile chiral auxiliary groups employed for the asymmetric synthesis of pharmaceuticals [47]. This exploration will include the use of this enzyme design in other chain reactions, and its comparison with traditional bioenzymatic systems in which an esterase and a protease are combined in one-pot, either in soluble form or co-immobilized preparations. That said, we have previously demonstrated that a *PluriZyme* supporting two different activities works as efficiently as when mixing separate enzymes supporting the two activities needed for the cascade reaction [20]. In both cases, the yields and conversions will depend on multiple factors, yet to be explored. For example, the separation of the two active sites within the protein scaffold in the case of the *PluriZyme* and the transfer of reaction intermediates between them; this can also occur between active centers of different enzymes in bi-enzymatic systems.

## Data Availability

The raw data supporting reported validation results can be found in the Supplementary Material (Raw Dataset).

## Supplementary Materials

The following are available online at https://www.mdpi.com/article/10.3390/ijms232113337/s1.
