# Supplementary material for: Design and Characterization of In-One Protease-Esterase PluriZyme
Source: Int J Mol Sci. 2022 Nov 1;23(21):13337. doi: 10.3390/ijms232113337 (PMC9655419; doi:10.3390/ijms232113337)
Supplement: Supplementary file 1 [file ijms-23-13337-s001.zip › ijms-1982724-supplementary.pdf]

Supporting Information

Design and characterization of in-one protease-esterase PluriZyme

Laura Fernandez-Lopez[1,†], Sergi Roda[2,†], Jose L. Gonzalez-Alfonso[1], Francisco J. Plou[1], Víctor Guallar[2,*] and Manuel Ferrer[1*]

Content

Table S1……… 2
Figure S1……... 2
Figure S2………3
Figure S3………3
Figure S4………4-7
Figure S5………7
Figure S6………8
Figure S7………9
Figure S8………9-10
Figure S9………10
Figure S10……..11
Raw Dataset…..12-18

Table S1: Absolute and relative number of accepted and total catalytic events (at 3.75 Å, 4.25 Å, and 5 Å) of EH_{1AB1C} from the local exploration for the different dipeptide substrates (A) and L-carnosine (B).

A)

Dipeptide substrate	Number of accepted catalytic events [3.75 Å]	Number of total catalytic events [3.75 Å]	Number of accepted catalytic events [4.25 Å]	Number of total catalytic events [4.25 Å]	Number of accepted catalytic events [5 Å]	Number of total catalytic events [5 Å]
AH	129 (0.265 %)	2121 (0.422 %)	443 (0.91 %)	7708 (1.533 %)	779 (1.6 %)	12349(2.455 %)
AQ	17 (0.04 %)	97 (0.019 %)	105 (0.248 %)	1709 (0.341 %)	421 (0.995 %)	5433 (1.083 %)
DI	3 (0.012 %)	6 (0.001 %)	68 (0.279 %)	1103 (0.22 %)	144 (0.59 %)	1597 (0.318 %)
EA	14 (0.034 %)	101 (0.02 %)	584 (1.416 %)	2887 (0.571 %)	1200 (2.91 %)	11398 (2.255 %)
FF	0	0	7 (0.025 %)	58 (0.012 %)	41 (0.145 %)	1468 (0.296 %)
KA	5 (0.013 %)	10 (0.002 %)	412 (1.093 %)	2840 (0.581 %)	1197 (3.177 %)	11745 (2.402 %)
LA	14 (0.033 %)	77 (0.015 %)	777 (1.858 %)	3748 (0.74 %)	1163 (2.781 %)	7051 (1.391 %)
LL	0	0	17 (0.044 %)	124 (0.025 %)	67 (0.172 %)	621 (0.124 %)
NV	0	0	7 (0.018 %)	49 (0.01 %)	371 (0.935 %)	3211 (0.638 %)
PF	58 (0.171 %)	1294 (0.259 %)	495 (1.456 %)	6355 (1.27 %)	657 (1.933 %)	7984 (1.596 %)
QQ	20 (0.065 %)	233 (0.047 %)	128 (0.415 %)	2656 (0.536 %)	295 (0.955 %)	4529 (0.914 %)
RG	9 (0.029 %)	36 (0.007 %)	171 (0.553 %)	2301 (0.465 %)	1016 (3.286 %)	9615 (1.941 %)
SW	47 (0.147 %)	1267 (0.256 %)	444 (1.393 %)	4699 (0.949 %)	513 (1.609 %)	5177 (1.045 %)
TM	38 (0.095 %)	570 (0.112 %)	404 (1.014 %)	7220 (1.418 %)	635 (1.594 %)	10478 (2.057 %)
YN	20 (0.081 %)	297 (0.06 %)	56 (0.227 %)	838 (0.17 %9	72 (0.292 %)	950 (0.192 %)
YY	0	0	0	0	0	0

B)

Dipeptide substrate	Number of accepted catalytic events [3.75 Å]	Number of total catalytic events [3.75 Å]	Number of accepted catalytic events [4.25 Å]	Number of total catalytic events [4.25 Å]	Number of accepted catalytic events [5 Å]	Number of total catalytic events [5 Å]
L-carnosine	26 (0.102 %)	433 (0.098 %)	166 (0.654 %)	2344 (0.531 %)	425 (1.675 %)	5082 (1.151 %)

Table S2: predicted $\Delta\Delta G_{(mut\text{-}WT)}$ of the $EH1_{AB1C}$ and its alternative calculated using the module of thermodynamic stability from HotSpot Wizard (see reference [45]) in both $EH1_A$ and EH_{1AB1} crystal structures.

Variant	$\Delta\Delta G_{(mut\text{-}WT)}$ [5JD4]	$\Delta\Delta G_{(mut\text{-}WT)}$ [6RB0]
EH_{1AB1C} (L24C)	6	4.2
EH_{1AB1C*} (L24C/V36H)	6.8	31.4

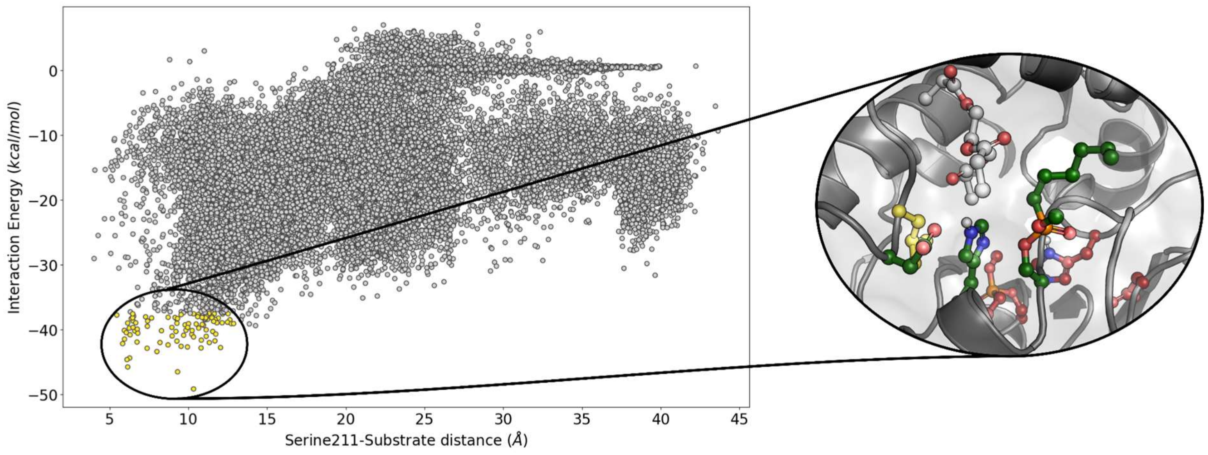

Figure S1. Global exploration of the potential hydrolase sites in the EH_{1AB1} structure with the methyl hydrogen (R)-hexylphosphonate inhibitor bound to both catalytic serine residues. Accepted PELE steps around, what we called site C, are highlighted in the energetic profile with a yellow color. On the right, we represent a binding pose of the probe ester in site C. The main active site has the C atoms stained in maroon, the artificial active site has them stained in dark green, and the potential residue to mutate to cysteine to add a protease site has them stained in yellow. The energy profile was created with the Matplotlib library (see reference [28]).

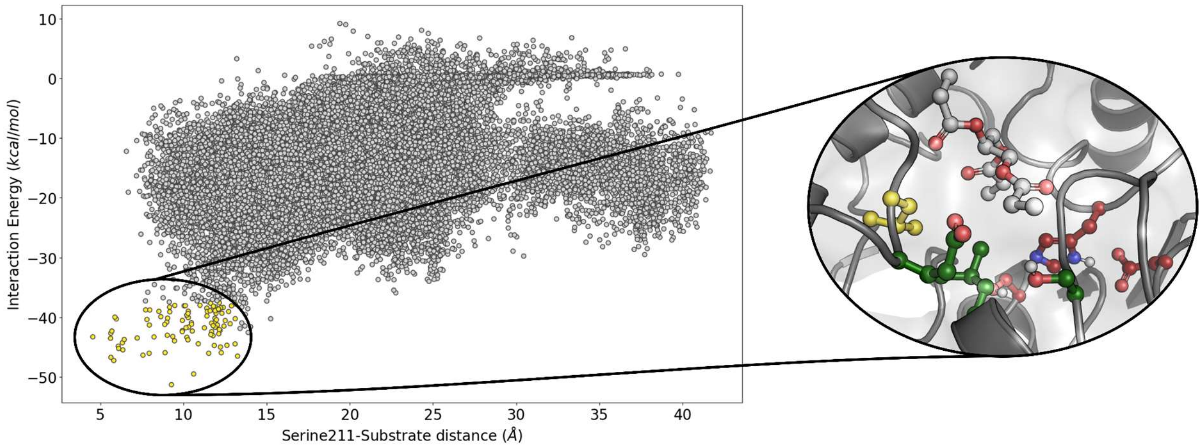

Figure S2. Global exploration of the potential hydrolase sites in the EH_{1A} structure. Accepted PELE steps around, what we called site C, are highlighted in the energetic profile with a yellow color. On the right, we represent a binding pose of the probe ester in site C. The main active site has the C atoms stained in maroon, the artificial active site has them stained in dark green, and the potential residue to mutate to cysteine to add a protease site has them stained in yellow. The energy profile was created with the Matplotlib library (see reference [28]).

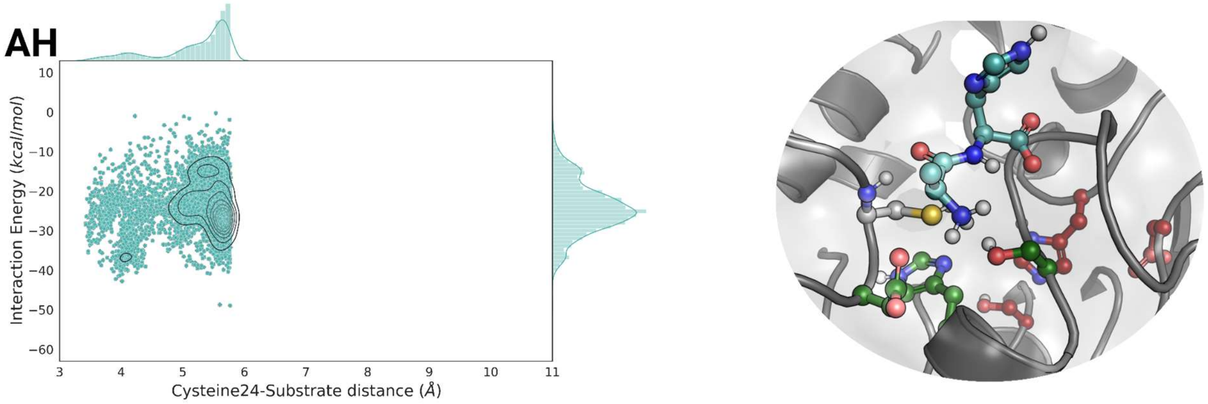
AH
Interaction Energy (kcal/mol)
Cysteine24-Substrate distance (Å)

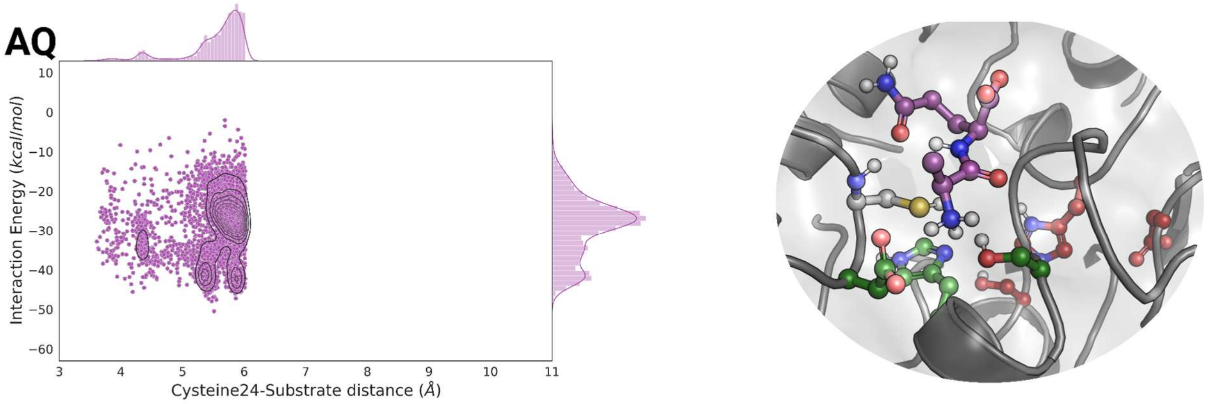
AQ
Interaction Energy (kcal/mol)
Cysteine24-Substrate distance (Å)

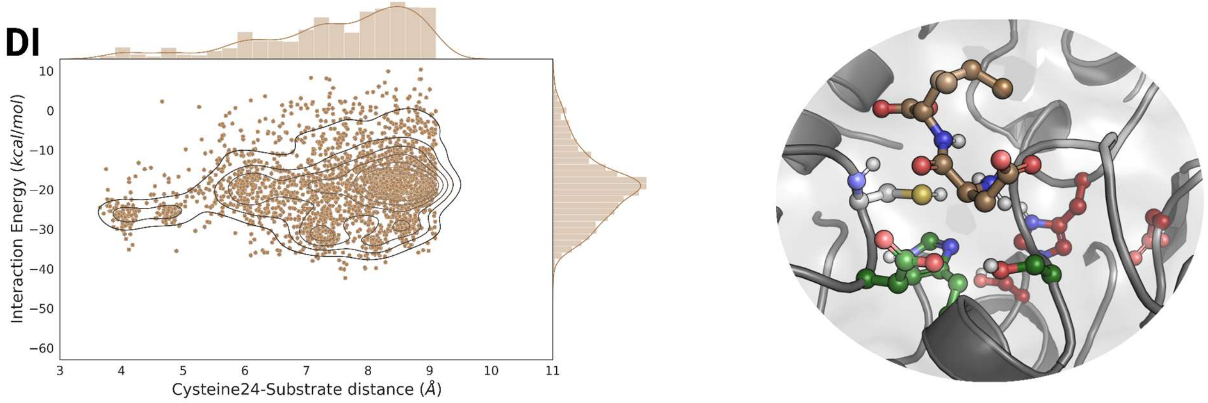
DI
Interaction Energy (kcal/mol)
Cysteine24-Substrate distance (Å)

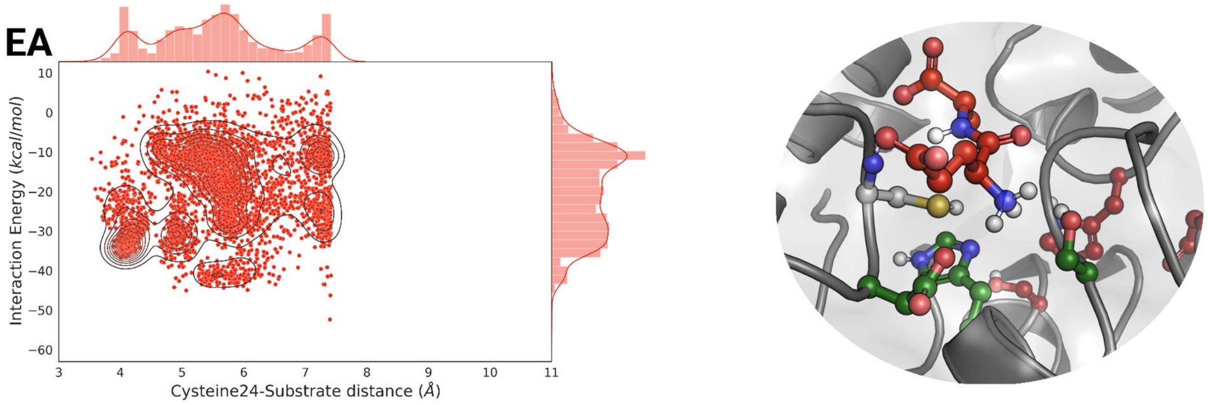
EA
Interaction Energy (kcal/mol)
Cysteine24-Substrate distance (Å)

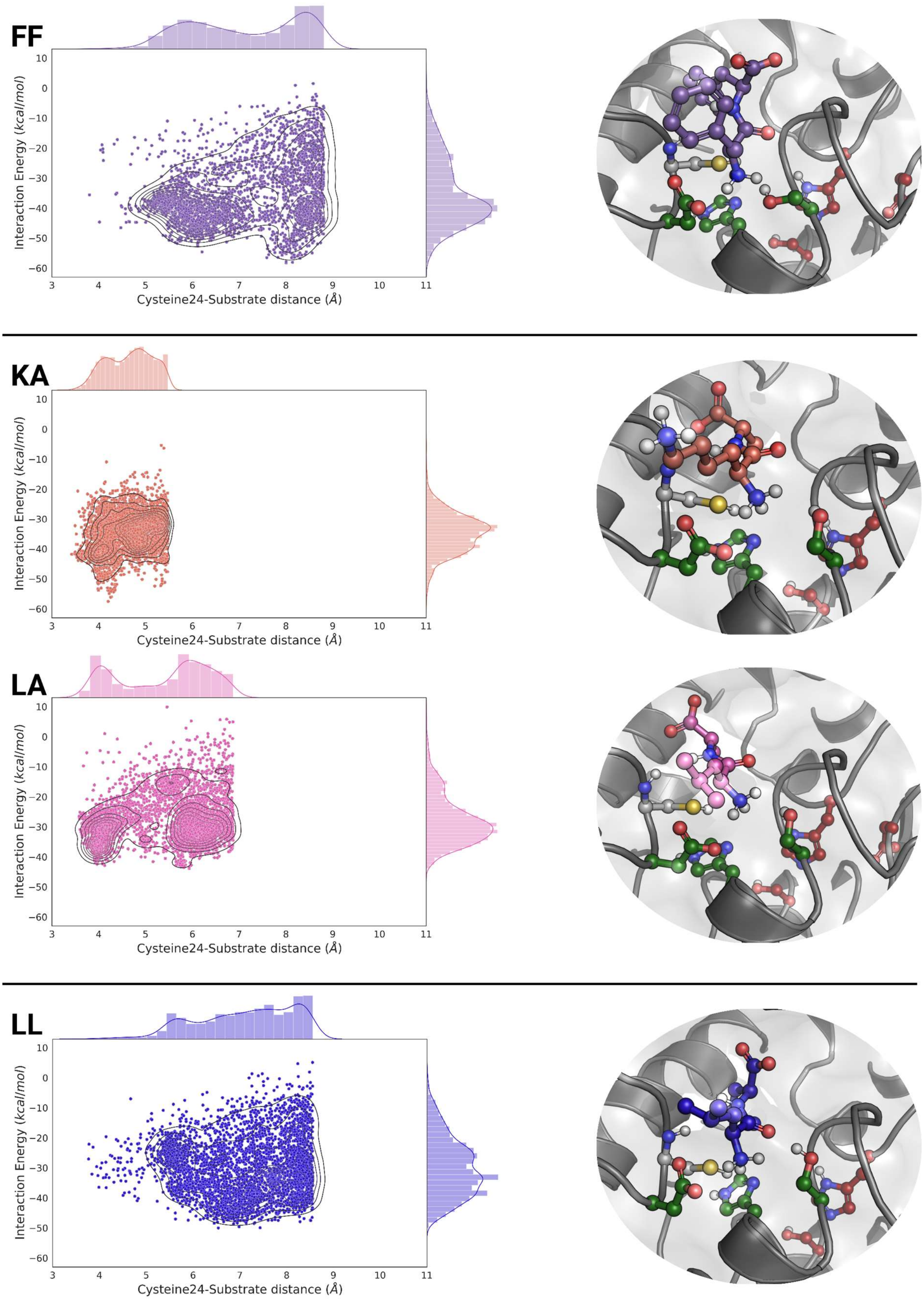

FF
Interaction Energy (kcal/mol)
10 0 −10 −20 −30 −40 −50 −60
3 4 5 6 7 8 9 10 11
Cysteine24-Substrate distance (Å)
KA
Interaction Energy (kcal/mol)
10 0 −10 −20 −30 −40 −50 −60
3 4 5 6 7 8 9 10 11
Cysteine24-Substrate distance (Å)
LA
Interaction Energy (kcal/mol)
10 0 −10 −20 −30 −40 −50 −60
3 4 5 6 7 8 9 10 11
Cysteine24-Substrate distance (Å)
LL
Interaction Energy (kcal/mol)
10 0 −10 −20 −30 −40 −50 −60
3 4 5 6 7 8 9 10 11
Cysteine24-Substrate distance (Å)

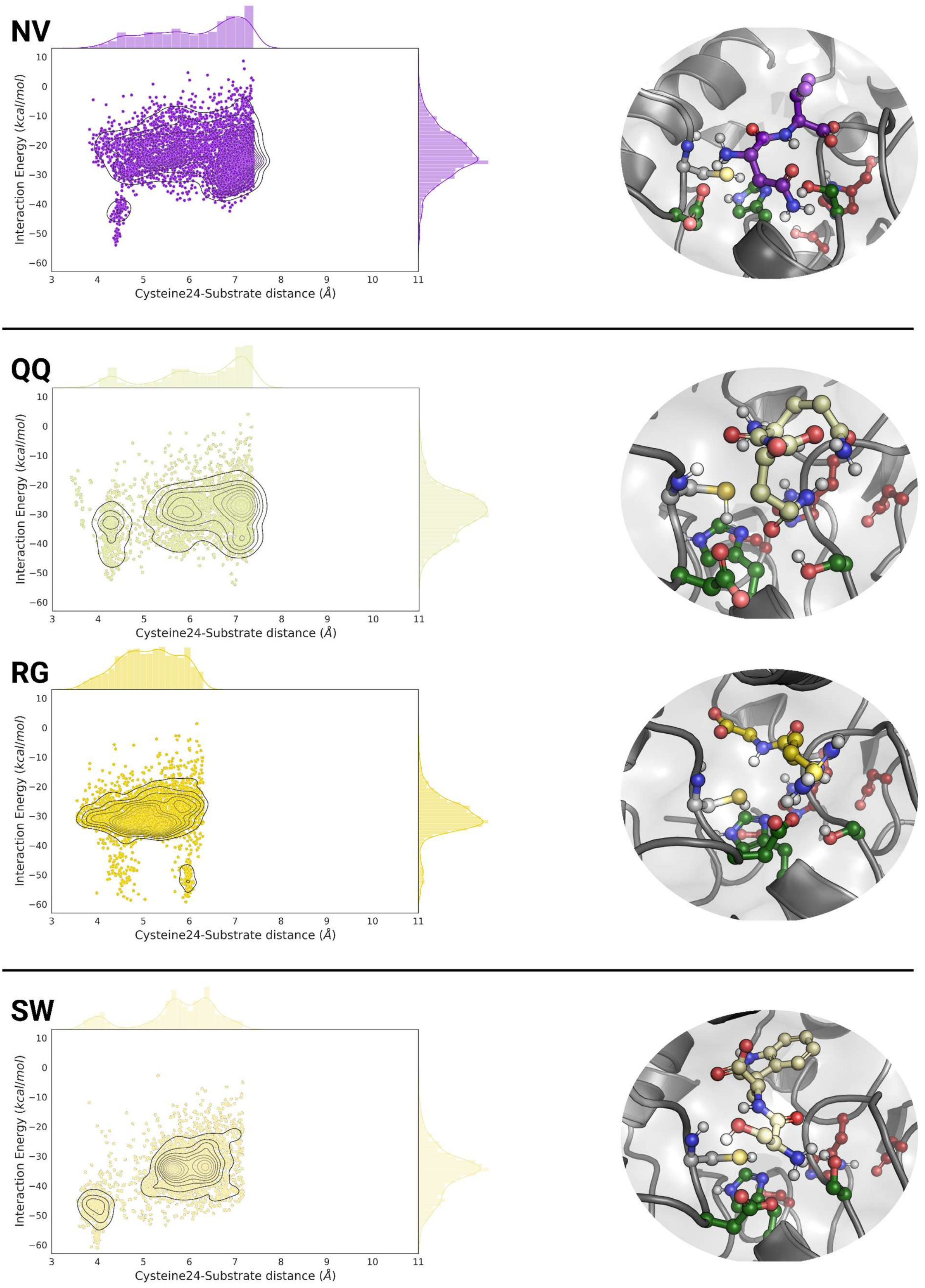

NV
QQ
RG
SW
Interaction Energy (kcal/mol)
Cysteine24-Substrate distance (Å)
10
0
−10
−20
−30
−40
−50
−60
3
4
5
6
7
8
9
10
11

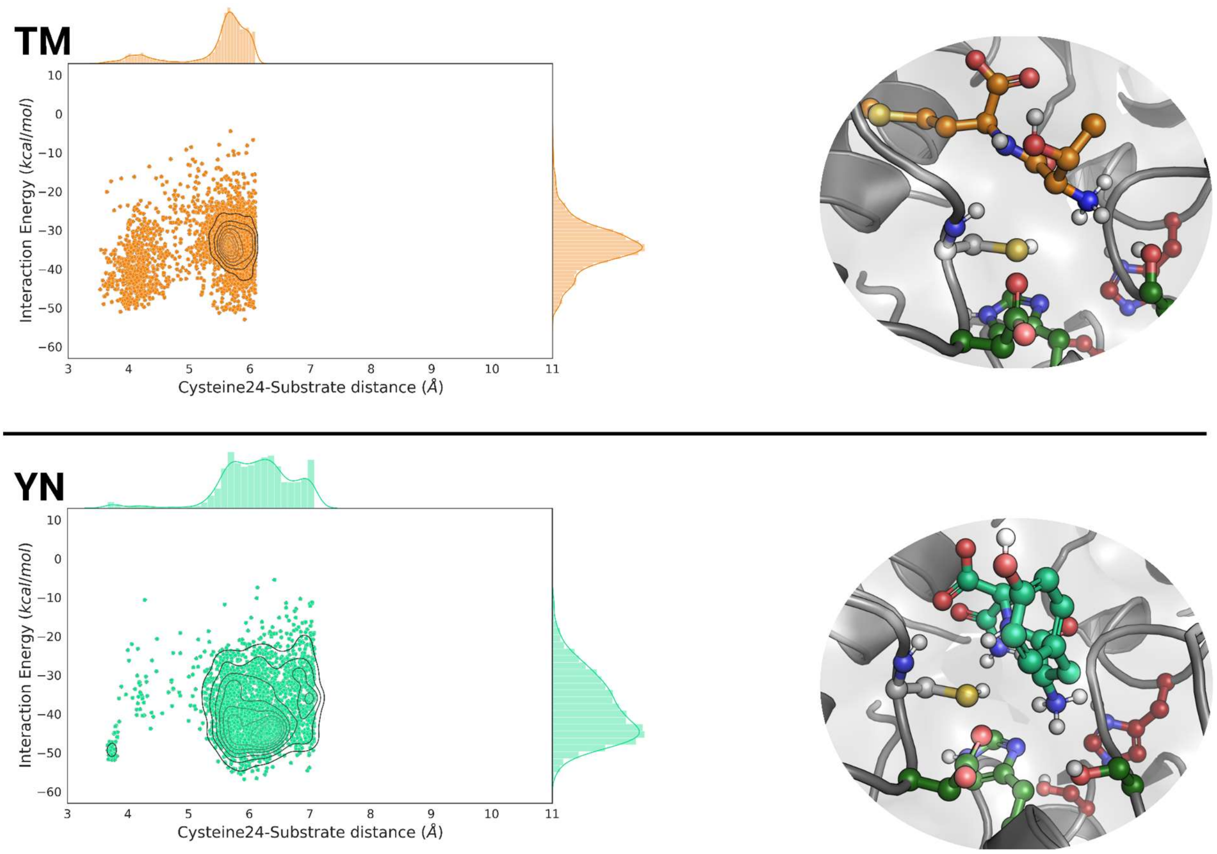

Figure S3. EH1AB1C density plots of the distribution of the catalytic cysteine-substrate distance against the interaction energy for the other simulated dipeptide substrates. Only the 10% lowest percentile regarding the distance is shown. On the right, we represent a catalytic pose of the dipeptide substrate in the protease site. The main active site has the C atoms stained in maroon, the artificial active site has them stained in dark green, the cysteine residue from the protease site has them stained in yellow, and each substrate has them stained in a particular color. The energy profiles were created with the Matplotlib library (see reference [28]).

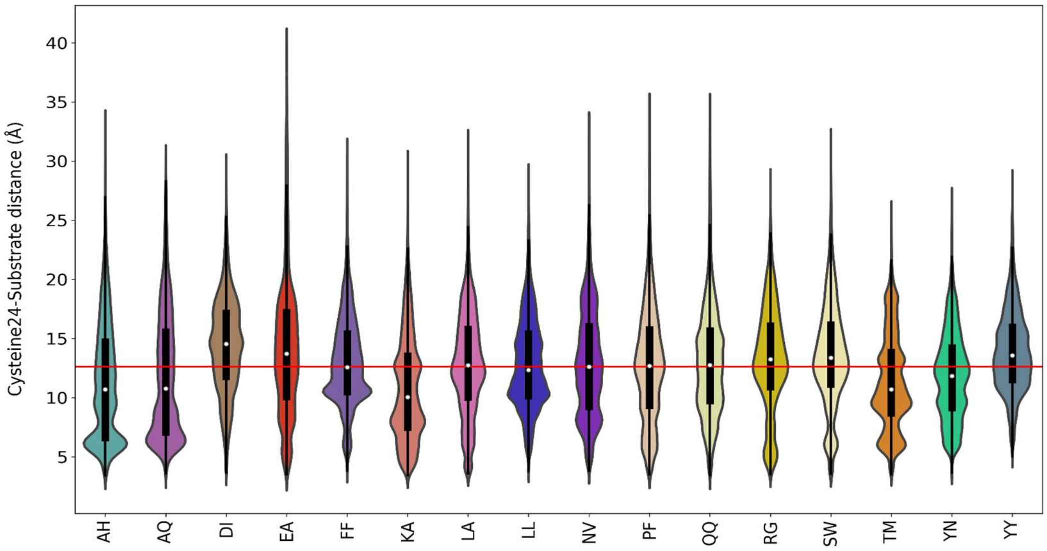

Figure S4. Violin plot representing the cysteine-substrate distance along all the accepted PELE steps from the local explorations for the different dipeptide substrates against EH1AB1C. The red line indicates the average value of the metric in all simulations. The figure was created with the Matplotlib library (see reference [28]).

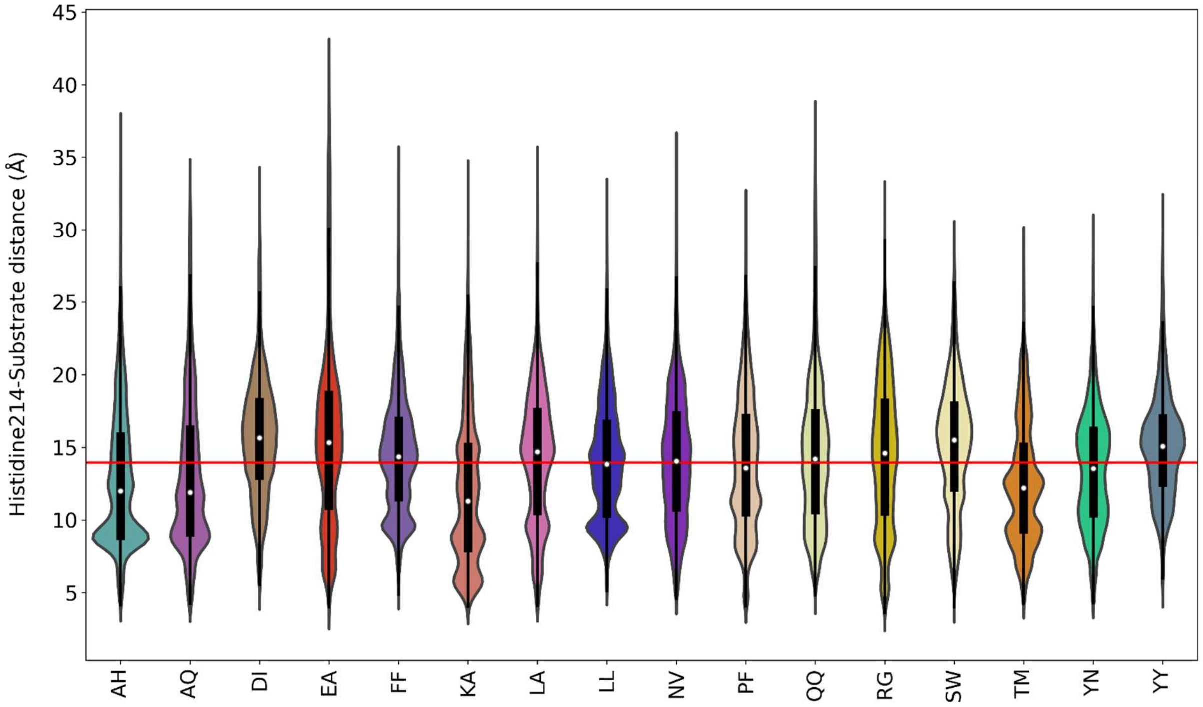

Figure S5. Violin plot representing the histidine-substrate distance along all the accepted PELE steps from the local explorations for the different dipeptide substrates against EH1AB1C. The red line indicates the average value of the metric in all simulations. The figure was created with the Matplotlib library (see reference [28]).

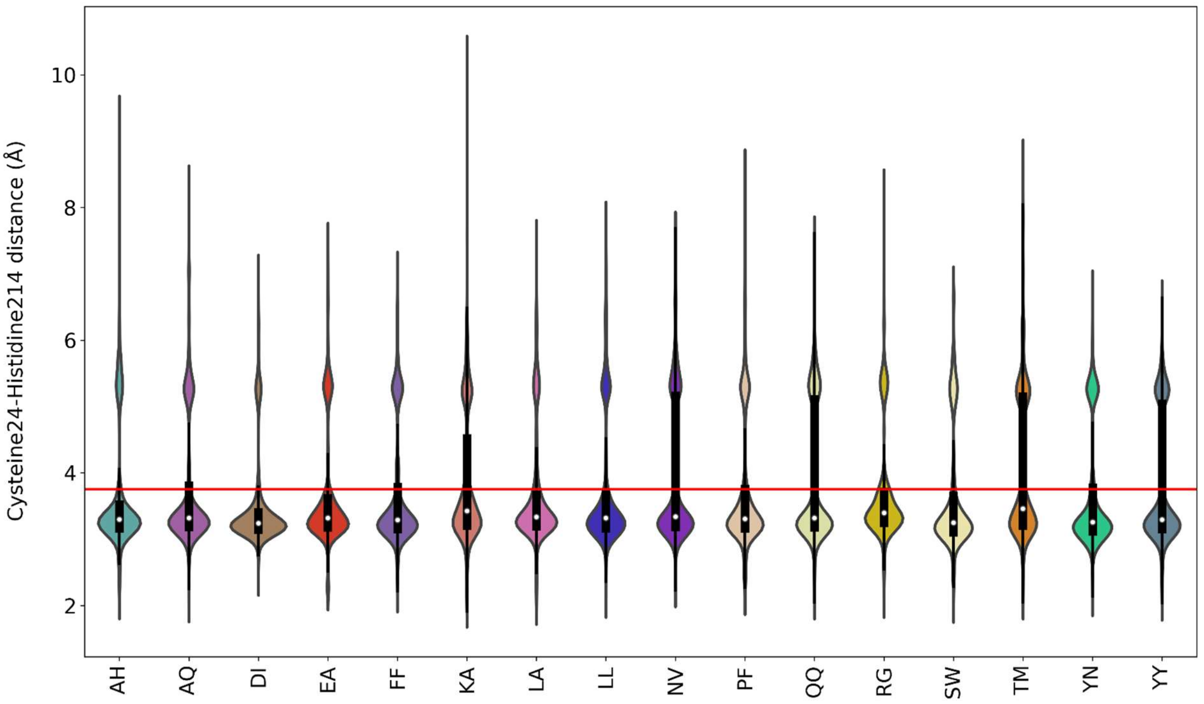

Figure S6. Violin plot representing the cysteine-histidine distance along all the accepted PELE steps from the local explorations for the different dipeptide substrates against EH1AB1C. The red line indicates the average value of the metric in all simulations. The figure was created with the Matplotlib library (see reference [28]).

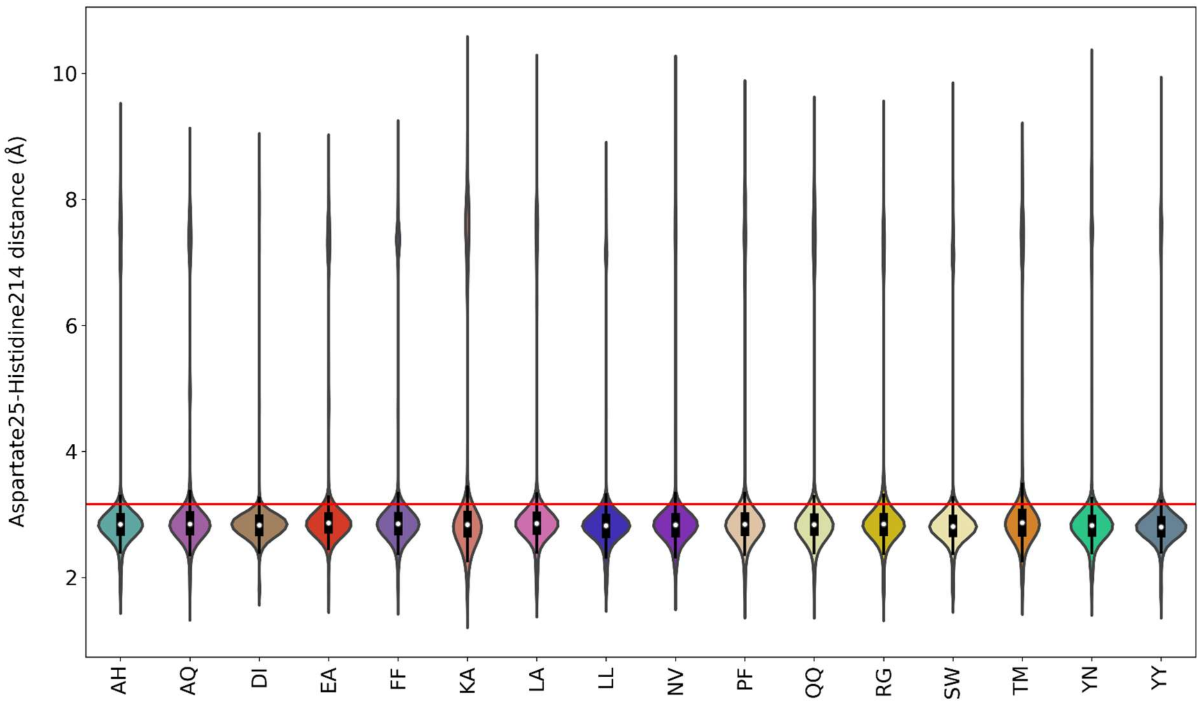

Figure S7. Violin plot representing the aspartate-histidine distance along all the accepted PELE steps from the local explorations for the different dipeptide substrates against EH1AB1C. The red line indicates the average value of the metric in all simulations. The figure was created with the Matplotlib library (see reference [28]).

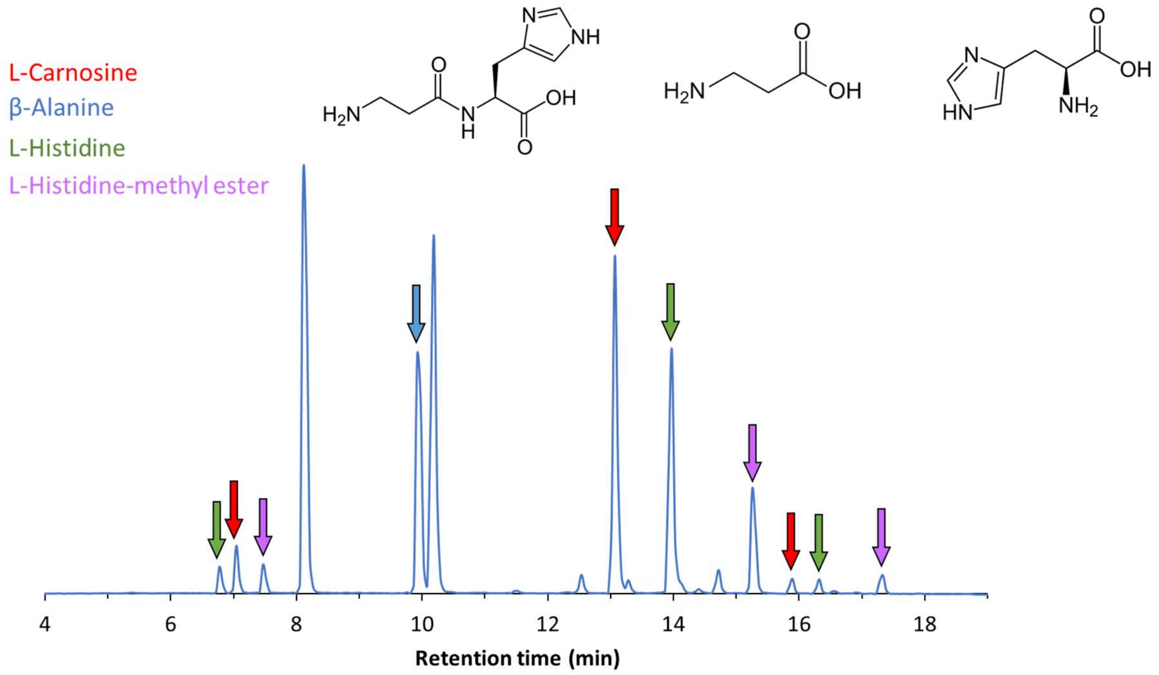

Figure S8. HPLC chromatograms representing the elution time of all substrates, intermediates and final products identified. As shown after derivatization some of the chemicals elute at different times (demonstrated by analysing each single chemical; not shown) and for the calculation of the concentration and conversion for each of them, the areas of each peak representing each chemical, were considered.

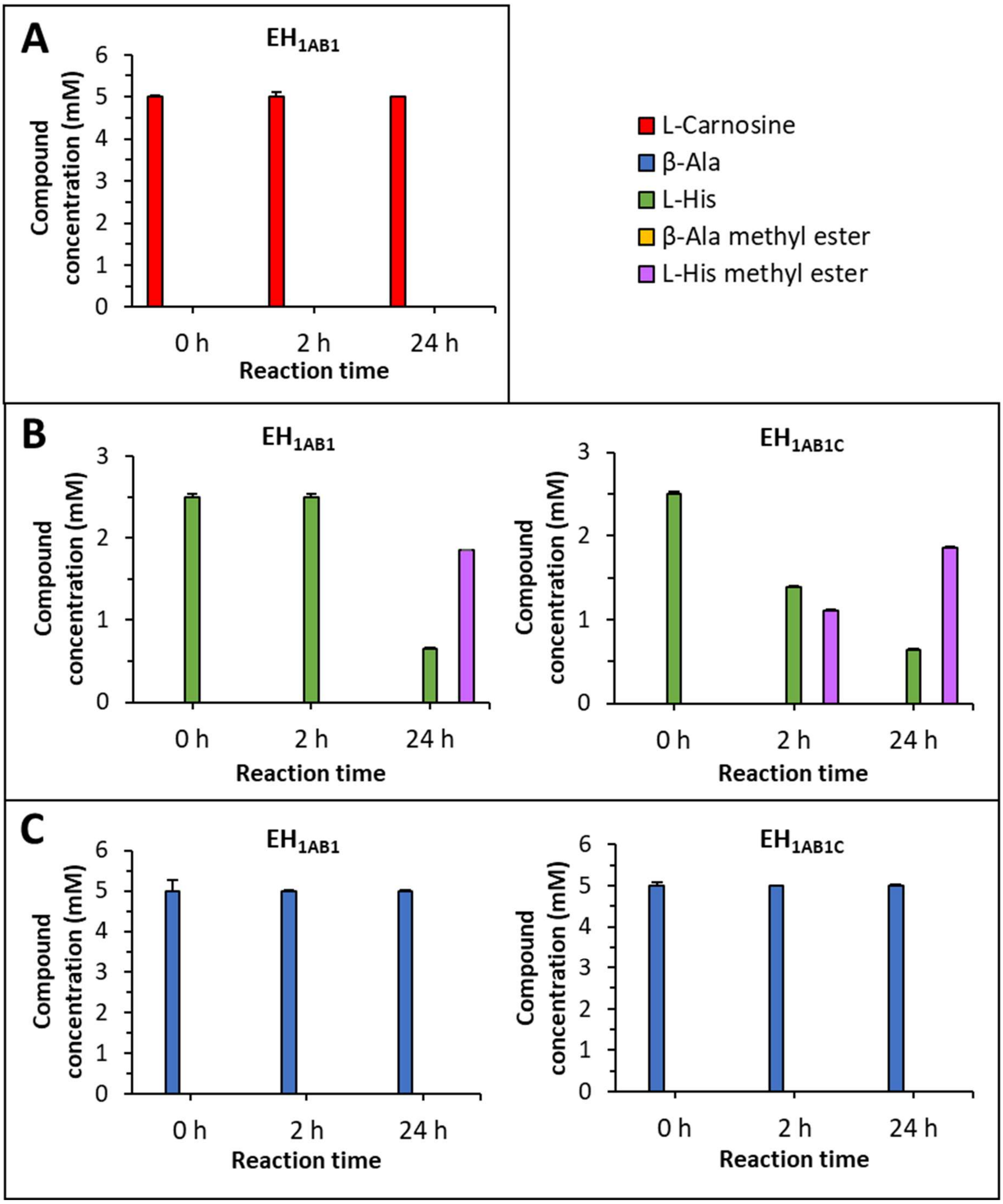

Figure S9. Concentrations of substrates and products obtained for the conversion of L-carnosine (A), L-histidine (B) and β-alanine (C) with whole cells expressing EH_{1AB1} *PluriZyme* (left panels) or EH_{1AB1C} *PluriZyme* (right panels) in the presence of methanol. The figure was created using SigmaPlot 14.0 software. As can be seen in panel A, the original EH_{1AB1} *PluriZyme* does not hydrolyse L-carnosine oes not esterify it. The results in panels B and C demonstrate that both the original EH_{1AB1} *PluriZyme* and the mutant EH_{1AB1C} PluriZyme esterify L-histidine, but not β-alanine.

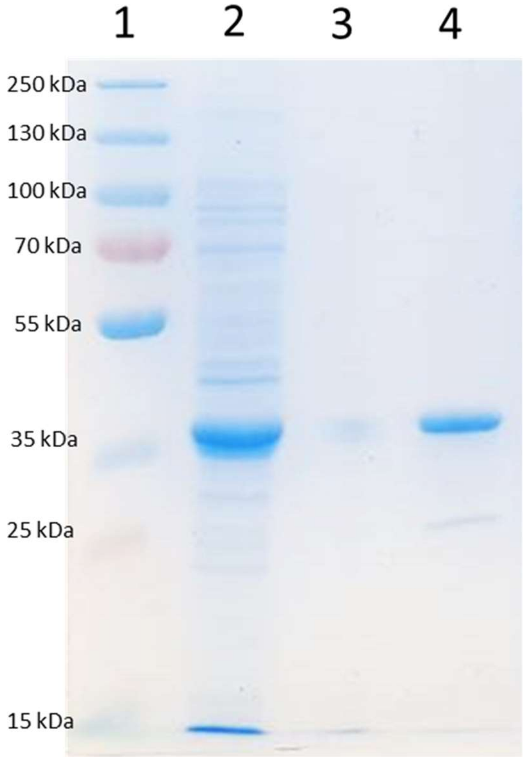

Figure S10. SDS-PAGE gel showing steps of EH_{1AB1C} purification. Twelve percent SDS-PAGE gel stained with Coomassie blue. Lane 1 contains molecular weight markers. Lane 2 contains whole cell lysate of *E. coli* expressing soluble protein. Lane 3 shows the whole cell lysate of *E. coli* expressing insoluble protein. Lane 4 shows the His-tagged protein and demonstrates protein purity of >95%.

(A)

pH	Fluorescence 485/20,528/20	
	EH1AB1C	
3	4	8
3,5	12	14
4	20	22
4,5	23	30
5	30	35
5,5	68	75
6	122	105
6,5	133	133
7	174	180
7,5	145	146
8	115	132
8,5	109	122

pH	Relative activity (%) EH1AB1C (average % and SD)	
3	3,4	1,6
3,5	7,4	0,8
4	11,9	0,8
4,5	15	2,8
5	18,4	2
5,5	40,4	2,8
6	64,1	6,8
6,5	75,1	0
7	100	2,4
7,5	82,2	0,4
8	69,8	6,8
8,5	65,3	5,2

(B)

T (°C)	Absorbance at 440 nm	
	EH1AB1C	
20	0,024	0,027
30	0,054	0,055
40	0,076	0,067
50	0,096	0,096
60	0,135	0,134
70	0,143	0,141
75	0,137	0,147
80	0,138	0,142
85	0,128	0,124
90	0,0112	0,0112

T (°C)	Relative activity (%) (average % and SD)	
20	18	1,5
30	38,4	0,5
40	50,4	4,5
50	67,6	0
60	94,7	0,5
70	100	1
75	100	5
80	98,6	2
85	88,7	2
90	7,8	0

(C)

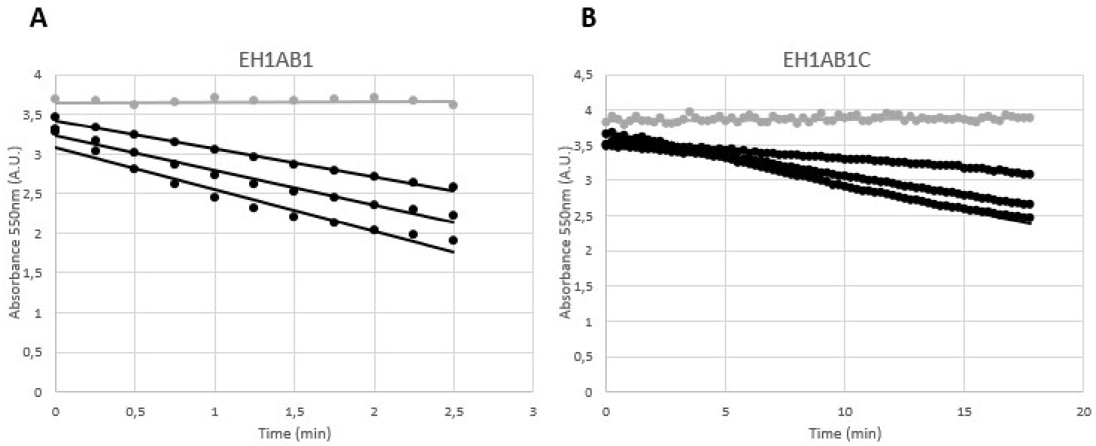

(D)

- Hydrolysis (buffer, no methanol) raw data (Figure 6A), in the presence of EH_{1AB1C}.

	Area - Average			
**Reaction time (h)**	Carnosine	β-Ala	His	His-methyl ester
0	66045886.33	0	0	0
1	56405937.67	9510840.333	12435133.33	0
2	20673479.67	18079872	26227694	0
24	5599927	34623204	56609214.33	0

	Area - Deviation			
**Reaction time (h)**	Carnosine	β-Ala	His	His-methyl ester
0	925255.158	0	0	0
1	4081705.52	129504.134	1999902.55	0
2	3855347.19	468065.626	3379406.28	0
24	1165884.48	620605.567	620605.567	0

	Sample concentration (µM)					
**Reaction time (h)**	Carnosine	β-Ala	His	His-methyl ester	Total	Total (residues)
0	417.6392355	0	0	0	417.6392355	835.2784709
1	356.6813013	124.2711031	103.7705251	0	584.7229295	941.4042308
2	130.7281456	236.2362902	218.868709	0	585.8331447	716.5612903
24	35.41097502	452.3957509	472.4008773	0	960.2076032	995.6185782

	SD (µM)			
**Reaction time (h)**	Carnosine	β-Ala	His	His-methyl ester
0	5.850824	0	0	0
1	25.8105457	1.69213456	16.6890802	0
2	24.3791755	6.1158667	28.2009654	0
24	7.37243649	8.10899308	5.17892039	0

Sample concentration in reaction medium (mM) -Normalized to (5 mM carnosine)					
**Reaction time (h)**	Carnosine	β-Ala	His	His-methyl ester	Total
0 h	5.000	0.000	0.000	0	5.000
1 h	3.789	1.320	1.102	0	6.211
2 h	1.824	3.297	3.054	0	8.176
24 h	0.356	4.544	4.745	0	9.644

SD mM				
**Reaction time (h)**	Carnosine	β-Ala	His	His-methyl ester
0	0.035	0.000	0.000	0.000
1	0.137	0.018	0.177	0.000
2	0.170	0.085	0.394	0.000
24	0.037	0.081	0.052	0.000

- Cascade reaction (with metanol) raw data (Figure 6B), in the presence of EH_{1AB1C}.

	Area - Average			
**Reaction time (h)**	Carnosine	β-Ala	His	His-methyl ester
0	72916723.33	0	0	0
1	55722512.67	9406029	7916764	4090492
2	21611397	19710882	17615182.33	8929280.333
24	4923047	34745890.67	16872401.67	26284110.33

	Area - Deviation			
**Reaction time (h)**	Carnosine	β-Ala	His	His-methyl ester
0	11167492.4	0	0	0
1	6726467.34	34811.0622	1241768.93	844628.1463
2	4946823.13	264351.172	2486694.35	2122136.349
24	1081449.47	369638.206	369638.206	5926748.901

	Sample concentration (μM)						
**Reaction time (h)**	Carnosine	β-Ala	His	His-methyl ester	Total	Total (residues	Total His+ His-ME
0	461.0867728	0	0	0	461.0867728	922.1735455	0
1	352.3596832	122.9016111	66.06497376	34.13493779	575.4612058	893.6859513	100.1999115
2	136.6590385	257.5474893	146.997758	74.51436861	615.7186544	677.8633242	221.5121266
24	31.13074408	453.9988066	140.7992929	219.3395002	845.2683439	657.0595877	360.1387932

	SD (μM)			
**Reaction time (h)**	Carnosine	β-Ala	His	His-methyl ester
0	70.6173123	0	0	0
1	42.53462	0.45485036	10.3624956	7.048376877
2	31.2810918	3.45408088	20.7513319	17.70911476
24	6.83851415	4.82978853	3.08461113	49.45840379

Sample concentration in reaction medium (mM) -Normalized to (5 mM carnosine)					
**Reaction time (h)**	L- Carnosine	β-Ala	His	His-methyl ester	Total
0 h	5.000	0.000	0.000	0.000	5.000
1 h	3.943	1.229	0.810	0.419	6.401
2 h	2.016	2.575	1.709	0.866	7.167
24 h	0.474	4.540	1.775	2.765	9.554

SD mM				
**Reaction time (h)**	Carnosine	β-Ala	His	His-methyl ester
0	0.383	0.000	0.000	0.000
1	0.238	0.005	0.104	0.070
2	0.231	0.035	0.208	0.177
24	0.052	0.048	0.031	0.495

(E)

- Cascade reaction (with metanol) raw data, in the presence of EH_{1AB1} (Figure S9A).

	Area - Average			
Reaction time (h)	Carnosine	β-Ala	His	His-methyl ester
0	74291435,33	0	0	0
2	62687474,33	0	0	0
20	52139788	0	0	0

	Area - Deviation				
Reaction time (h)	Carnosine	β-Ala	His	β-Ala methyl ester	His-methyl ester
0	609950,783	0	0	0	0
2	3036089,87	0	0	0	0
20	324015,542	0	0	0	0

	Sample concentration (µM)					
Reaction time (h)	Carnosine	β-Ala	His	His-methyl ester	β-Ala methyl ester	Total
0	469,779724	0	0	0	0	469,779724
2	396,4024151	0	0	0	0	396,4024151
20	329,7044283	0	0	0	0	329,7044283

	SD (µM)				
Reaction time (h)	Carnosine	β-Ala	His	β-Ala methyl ester	His-methyl ester
0	3,85700598	0	0		0
2	19,1986257	0	0		0
20	2,04890283	0	0		0

Sample concentration in reaction medium (mM) -Normalized to (5 mM carnosine)					
Reaction time (h)	L-Carnosine	β-Ala	L-His	L-His methyl ester	β-Ala methyl ester	Total
0	5,000	0,000	0,000	0		5,000
2	5,000	0,000	0,000	0		5,000
20	5,000	0,000	0,000	0		5,000

SD mM					
Reaction time (h)	Carnosine	β-Ala	His	β-Ala methyl ester	His-methyl ester
0	0,021	0,000	0,000	0	0,000
2	0,121	0,000	0,000	0	0,000
20	0,016	0,000	0,000	0	0,000

- L- Histidine reaction (with metanol) raw data in the presence of EH_{1AB1} (Figure S9B).

	Area - Average			
Reaction time (h)	Carnosine	β-Ala	His	His-methyl ester
0	0	0	35535189.67	0
2	0	0	31907237	0
24	0	0	12867910.33	36645382

	Area - Deviation			
Reaction time (h)	Carnosine	β-Ala	His	His-methyl ester
0	0	0	575075.85	0
2	0	0	475937.293	0
24	0	0	392832.675	189697.3025

	Sample concentration (µM)					
Reaction time (h)	Carnosine	β-Ala	His	His-methyl ester	Total	Total (residuos)
0	0	0	296.5392644	0	296.5392644	296.5392644
2	0	0	266.2641927	0	266.2641927	266.2641927
24	0	0	107.3820261	305.8037602	413.1857863	413.1857863

	SD (µM)			
Reaction time (h)	Carnosine	β-Ala	His	His-methyl ester
0	0	0	4.79897732	0
2	0	0	3.97167135	0
24	0	0	3.27816774	1.583013881

Sample concentration in reaction medium (mM) -Normalized to (2.5 mM His)					
Reaction time (h)	L-Carnosine	β-Ala	L-His	L- His-methyl ester	Total
0 h	0.000	0.000	2.500	0.000	2.500
2 h	0.000	0.000	2.500	0.000	2.500
24 h	0.000	0.000	0.650	1.850	2.500

SD mM				
Reaction time (h)	Carnosine	β-Ala	His	His-methyl ester
0	0.000	0.000	0.040	0.000
2	0.000	0.000	0.037	0.000
24	0.000	0.000	0.020	0.010

- - Histidine reaction (with metanol) raw data in the presence of EH_{1AB1C} (Figure S9B).

	Area - Average			
Reaction time (h)	Carnosine	β-Ala	His	His-methyl ester
0	0	0	35111999	0
2	0	0	25123322.67	19989914.67
24	0	0	14500647.67	41832287.33

	Area - Deviation			
Reaction time (h)	Carnosine	β-Ala	His	His-methyl ester
0	0	0	401282.111	0
2	0	0	282187.094	162377.1727
24	0	0	87384.3772	297615.2006

	Sample concentration (µM)					
Reaction time (h)	Carnosine	β-Ala	His	His-methyl ester	Total	Total (residues)
0	0	0	293.0077608	0	293.0077608	293.0077608
2	0	0	209.652789	166.8147728	376.4675618	376.4675618
24	0	0	121.0071321	349.0882089	470.095341	470.095341

	SD (µM)			
Reaction time (h)	Carnosine	β-Ala	His	His-methyl ester
0	0	0	3.34867783	0
2	0	0	2.35483627	1.355028854
24	0	0	0.72921797	2.483582991

Sample concentration in reaction medium (mM) -Normalized to (2.5 mM His)					
Reaction time (h)	L-Carnosine	β-Ala	L-His	L- His-methyl ester	Total
0 h	0.000	0.000	2.500	0.000	2.500
2 h	0.000	0.000	1.392	1.108	2.500
24 h	0.000	0.000	0.644	1.856	2.500

SD mM				
Reaction time (h)	Carnosine	β-Ala	His	His-methyl ester
0	0.000	0.000	0.029	0.000
2	0.000	0.000	0.016	0.009
24	0.000	0.000	0.004	0.013

- β-Alanine reaction (with metanol) raw data in the presence of EH_{1AB1} (Figure S9C).

	Area - Average			
Reaction time (h)	Carnosine	β-Ala	His	His-methyl ester
0	0	38621021.33	0	0
2	0	34250592	0	0
24	0	35603589.33	0	0

	Area - Deviation			
Reaction time (h)	Carnosine	β-Ala	His	His-methyl ester
0	0	1955503.02	0	0
2	0	112858.066	0	0
24	0	33521.6922	0	0

Reaction time (h)	Sample concentration (µM) Carnosine	β-Ala	His	His-methyl ester	Total	Total (residues)
0	0	504.6322676	0	0	504.6322676	504.6322676
2	0	447.527106	0	0	447.527106	447.527106
24	0	465.2057195	0	0	465.2057195	465.2057195

Reaction time (h)	SD (µM) Carnosine	β-Ala	His	His-methyl ester
0	0	25.5511089	0	0
2	0	1.47463272	0	0
24	0	0.43800311	0	0

Sample concentration in reaction medium (mM) -Normalized to (5 mM Ala)					
Reaction time (h)	L-Carnosine	β-Ala	L-His	L- His-methyl este	Total
0 h	0.000	5.000	0.000	0.000	5.000
2 h	0.000	5.000	0.000	0.000	5.000
24 h	0.000	5.000	0.000	0.000	5.000

SD mM				
Reaction time (h)	Carnosine	β-Ala	His	His-methyl ester
0	0.000	0.253	0.000	0.000
2	0.000	0.016	0.000	0.000
24	0.000	0.005	0.000	0.000

- β-Alanine reaction (with metanol) raw data in the presence of EH_{1AB1C} (Figure S9C).

Reaction time (h)	Area - Average Carnosine	β-Ala	His	His-methyl ester
0	0	37663773.33	0	0
2	0	34222480	0	0
24	0	33938377.33	0	0

Reaction time (h)	Area - Deviation Carnosine	β-Ala	His	His-methyl ester
0	0	715050.418	0	0
2	0	67054.195	0	0
24	0	247115.117	0	0

Reaction time (h)	Sample concentration (µM) Carnosine	β-Ala	His	His-methyl ester	Total	Total (residues)
0	0	492.1246173	0	0	492.1246173	492.1246173
2	0	447.1597873	0	0	447.1597873	447.1597873
24	0	443.4476283	0	0	443.4476283	443.4476283

Reaction time (h)	SD (µM) Carnosine	β-Ala	His	His-methyl ester
0	0	9.34303396	0	0
2	0	0.87614748	0	0
24	0	3.22887011	0	0

Sample concentration in reaction medium (mM) -Normalized to (5 mM Ala)					
Reaction time (h)	L-Carnosine	β-Ala	L-His	L- His-methyl este	Total
0 h	0.000	5.000	0.000	0.000	5.000
2 h	0.000	5.000	0.000	0.000	5.000
24 h	0.000	5.000	0.000	0.000	5.000

SD mM				
Reaction time (h)	Carnosine	β-Ala	His	His-methyl ester
0	0.000	0.095	0.000	0.000
2	0.000	0.010	0.000	0.000
24	0.000	0.036	0.000	0.000

Raw Dataset. (A) Raw fluorescence data (corresponding to Figure 3A) after the hydrolysis of BODIBY FL casein by EH_{1AB1C}, at different pH values (corrected by background signal). Reaction conditions as described in Section 4.5. (B) Raw absorbance data (corresponding to Figure 3B) after the hydrolysis of azocasein by EH_{1AB1C}, at different temperatures. Reaction conditions as described in Section 4.5. (B) Representative time-course curve for the hydrolysis of glyceryl tripropionate by EH_{1AB1C} (corrected by background signal). Shown are the raw data (absorbance at 550 nm over time), corresponding to calculations of specific activity. Reaction conditions as described in Section 4.5. (A) Representative time-course curve for the hydrolysis of glyceryl tripropionate by EH_{1AB1C} andEH_{1AB1}. Shown are the raw data (absorbance at 550 nm over time), corresponding to calculations of specific activity. Reaction conditions as described in Section 4.5. (D)

Raw data and calculations corresponding to Figure 5 (cascade reaction with EH_{1AB1C}). (E) Raw data and calculation for the control cascade reaction in the presence of EH_{1AB1C}; as shown no reactions products were found.
